# Ureteral Involvement in Non-Hodgkin Lymphoma: A Rare Case Report With Diagnostic Challenges

**DOI:** 10.7759/cureus.108210

**Published:** 2026-05-03

**Authors:** Filip Milutinovic, Filip Z Mihajlovic, Dalibor Jovanovic, Vladan Markovic, Bojan Stojanovic, Djordje Todorovic, Djordje Djordjevic, Damnjan Pantic, Milena Ilic

**Affiliations:** 1 Department of Urology, University Clinical Center Kragujevac, Kragujevac, SRB; 2 Department of Surgery, Faculty of Medical Sciences, University of Kragujevac, Kragujevac, SRB; 3 Department of Forensic Medicine, Faculty of Medical Sciences, University of Kragujevac, Kragujevac, SRB; 4 Department of Pathology, University Clinical Center Kragujevac, Kragujevac, SRB; 5 Department of Pathology, Faculty of Medical Sciences, University of Kragujevac, Kragujevac, SRB; 6 Department of Radiology, University Clinical Center Kragujevac, Kragujevac, SRB; 7 Department of Radiology, Faculty of Medical Sciences, University of Kragujevac, Kragujevac, SRB; 8 Department of Surgery, University Clinical Center Kragujevac, Kragujevac, SRB; 9 Department of Research, Faculty of Medical Sciences, University of Kragujevac, Kragujevac, SRB

**Keywords:** differential diagnosis, diffuse large b-cell lymphoma, rare case, r-chop therapy, ureteral lymphoma

## Abstract

Ureteral involvement by diffuse large B-cell lymphoma (DLBCL) is an extremely rare clinical entity that can mimic primary urothelial carcinoma and pose a significant diagnostic challenge. A 70-year-old female patient initially presented to a urologist with renal colic. The patient denied fever, night sweats, unintentional weight loss, or prior hematologic disorders, and no peripheral lymphadenopathy was detected on physical examination. Multislice computed tomography (MSCT) revealed a ureteral mass, followed by cystoscopy with ureteral stent placement. Due to the strong clinical and radiological suspicion of upper tract malignancy, most likely ureteric urothelial carcinoma, radical nephroureterectomy was performed as the standard definitive management in the absence of a feasible preoperative tissue diagnosis. Definitive diagnosis was established through pathohistological and immunohistochemical analysis, confirming diffuse large B-cell non-Hodgkin lymphoma (NHL), germinal center B-cell-like subtype, with a high Ki-67 proliferative index (~80%). Staging work-up demonstrated localized disease (clinical stage I with extranodal involvement) by the Ann Arbor classification, with no evidence of systemic dissemination. The patient was treated with standard Rituximab, Cyclophosphamide, Doxorubicin, Vincristine, and Prednisone (R-CHOP) immunochemotherapy, which was well tolerated. Early detection and timely initiation of therapy were associated with a favorable initial clinical course, as evidenced by clinical stabilization, absence of disease progression during follow-up, no early complications, and a good response to the R-CHOP regimen. This case highlights the extreme rarity of ureteral involvement by lymphoma, emphasizing the importance of considering lymphoma in the differential diagnosis of ureteral masses. It also underscores the critical role of histopathological evaluation in establishing an accurate diagnosis. Early recognition and appropriate treatment are essential for improving patient outcomes in such rare presentations.

## Introduction

Lymphomas are malignant disorders of the lymphocytic system that can occur in lymph nodes as well as in various extranodal locations, including the urinary tract. Diffuse large B-cell lymphoma (DLBCL) is the most common type of non-Hodgkin lymphoma (NHL) in adults and accounts for a significant proportion of all NHL cases; however, its occurrence in the urinary tract is extremely rare [1]. Epidemiological analyses of primary urinary tract DLBCL indicate that most cases involve the kidney and urinary bladder, whereas ureteral involvement accounts for only a very small fraction (<1%) of cases, highlighting the rarity of this site [2].

Ureteral lymphomas are mostly reported as individual cases and represent a diagnostic challenge because their clinical and radiological presentation may mimic primary urothelial tumors of the ureter, including carcinoma, often resulting in delayed diagnosis [3]. Literature report includes a case of primary B-cell lymphoma of the ureter in a young patient presenting with bilateral obstructive uropathy, in which histopathological confirmation was obtained, and treatment with Rituximab, Cyclophosphamide, Doxorubicin, Vincristine, and Prednisone (R-CHOP) immunochemotherapy resulted in a favorable outcome [3]. Another report describes malignant lymphoma of the ureter in an adult, emphasizing that although extremely rare, it should be considered in the differential diagnosis, particularly in patients with unexplained ureteral stenosis or a ureteral mass [4]. Furthermore, available studies suggest that primary ureteral DLBCL is an aggressive malignancy, while a combination of surgical intervention and systemic chemotherapy may significantly influence patient outcomes [1]. Analyses of patients with primary ureteral DLBCL indicate that kidney and bladder involvement is far more common than ureteral involvement; however, the histologic characteristics and treatment approaches are relevant for understanding the behavior of this disease in the urinary tract [1].

In addition to ureteral involvement, DLBCL can rarely occur in other parts of the urinary tract, such as the bladder, where it often presents with hematuria or other nonspecific symptoms, further emphasizing the difficulty of establishing a correct diagnosis without histological examination [5]. Due to the rarity of ureteral lymphoma and its ability to mimic urologic tumors, timely diagnostic evaluation, including detailed imaging, biopsy, and immunohistochemical testing, is essential for appropriate therapy [2,3]. This rarity also means that most available data are in the form of individual cases or small case series, making literature reviews and case reports valuable for educating clinicians and pathologists about this unusual disease presentation [4,5].

The objective of this study is to present a rare case of primary extranodal DLBCL arising in the ureter and to highlight the associated diagnostic challenges.

## Case presentation

**Figure 1:**
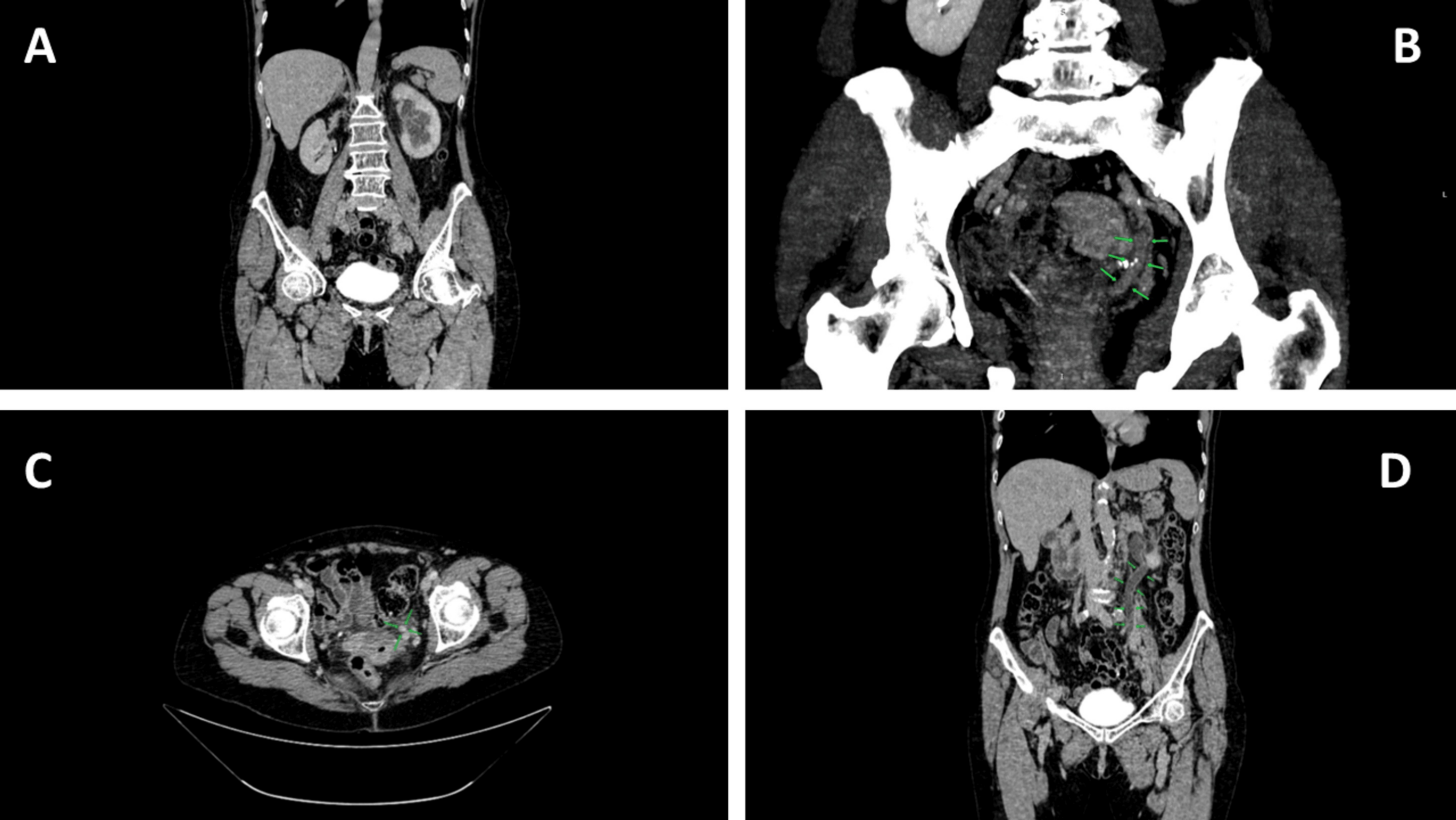
CT imaging demonstrating a ureteral tumor (A) Coronal CT image in the excretory phase showing dilatation of the left renal calyces. (B) Contrast-enhanced coronal CT image (maximum intensity projection) demonstrating a soft-tissue mass within the pelvic ureter (arrows). (C) Contrast-enhanced axial CT image in the nephrographic phase showing an enhancing soft-tissue mass within the left ureter (arrows). (D) Contrast-enhanced coronal CT image demonstrating a soft-tissue mass within the pelvic ureter (arrows).

A 70-year-old female patient initially presented to a urologist with renal colic. The patient denied fever, night sweats, unintentional weight loss, or prior hematologic disorders, and no peripheral lymphadenopathy was detected on physical examination. Urinalysis revealed microhematuria. Ultrasonography demonstrated grade II ureterohydronephrosis, after which the patient received sulfamethoxazole-trimethoprim 480 mg for five days with appropriate medical advice. However, follow-up examination showed no change in the findings. Plain abdominal radiography was subsequently performed, but did not reveal any pathological findings. This examination was included as part of the initial urological work-up in a patient presenting with flank pain and suspected urinary tract obstruction, primarily to exclude radiopaque urolithiasis. Subsequently, multislice computed tomography (MSCT) was performed, raising suspicion of a ureteral neoplasm (Figure 1).

**Figure 2:**
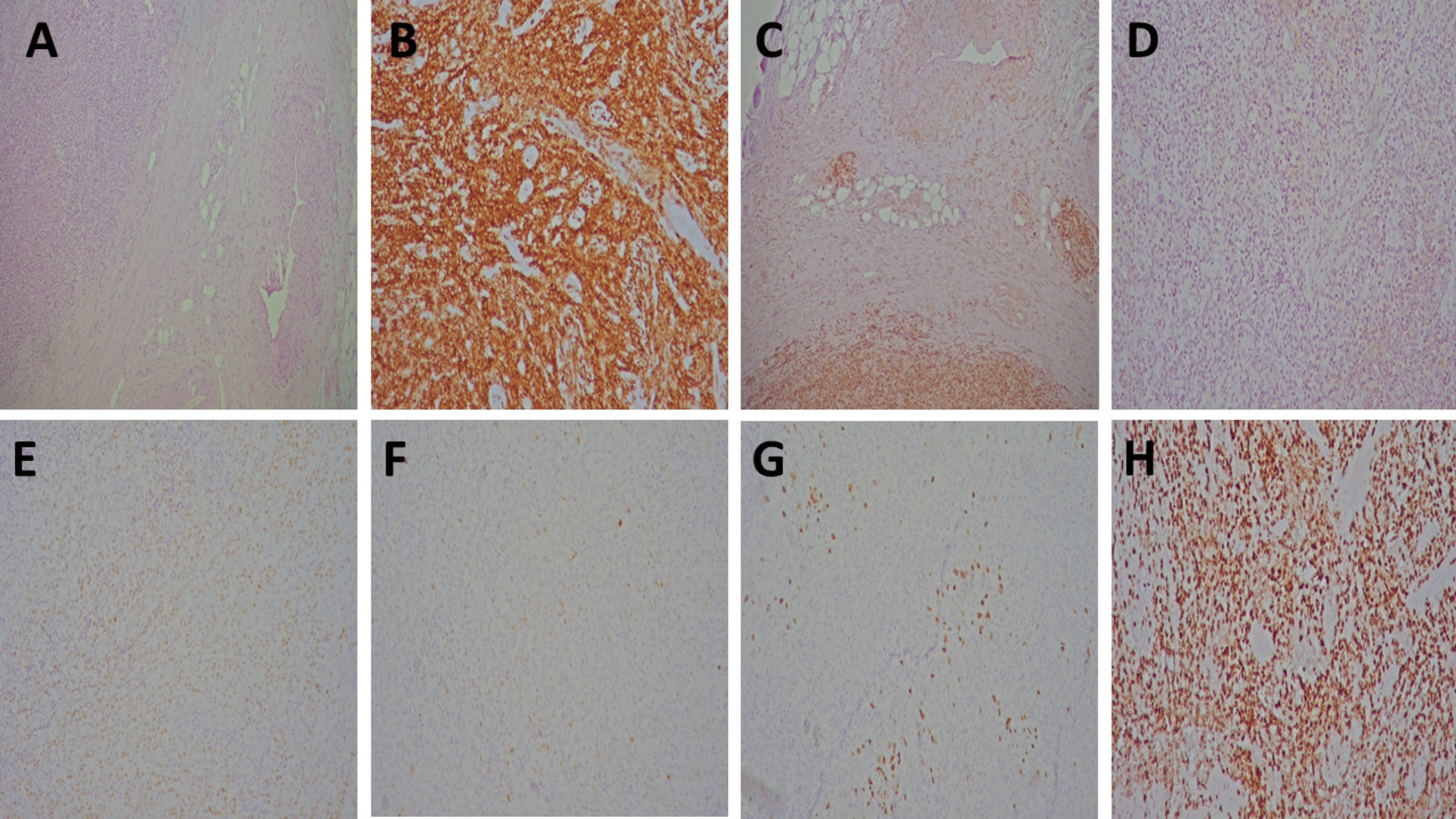
Histopathological and immunohistochemical features of the ureteral lymphoproliferative neoplasm A: Microscopic image of an H&E-stained tumor specimen of the ureter (histochemical analysis, original magnification 50×). B: Microscopic image showing expression of the LCA marker in B lymphocytes of a lymphoproliferative neoplasm (immunohistochemical analysis, original magnification 100×). C: Microscopic image showing expression of the CD79a marker in B lymphocytes of a lymphoproliferative neoplasm (immunohistochemical analysis, original magnification 100×). D: Microscopic image showing absence of CD10 expression in B lymphocytes of a lymphoproliferative neoplasm (immunohistochemical analysis, original magnification 100×). E: Microscopic image showing expression of the Bcl6 marker in B lymphocytes of a lymphoproliferative neoplasm (immunohistochemical analysis, original magnification 100×). F: Microscopic image showing partial expression of the MUM1 marker in B lymphocytes of a lymphoproliferative neoplasm (immunohistochemical analysis, original magnification 100×). G: Microscopic image showing expression of the CD5 marker in T lymphocytes of a lymphoproliferative neoplasm (immunohistochemical analysis, original magnification 100×). H: Microscopic image showing expression of the Ki67 marker in B lymphocytes of a lymphoproliferative neoplasm (immunohistochemical analysis, original magnification 100×). H&E: Hematoxylin and Eosin; LCA: leukocyte common antigen; CD: cluster of differentiation; Bcl: B-cell Lymphoma; MUM: multiple myeloma

For further evaluation, cystoscopy was performed, revealing a patent urethra and urinary bladder mucosa without pathological proliferations. Given the clearly visualized tumor of the left ureter, its size, and the associated urinary obstruction, a decision was made to perform left-sided radical nephroureterectomy. Due to the clinical and radiological suspicion of malignancy, with primary suspicion of ureteral urothelial carcinoma, radical nephroureterectomy was performed as the standard diagnostic and therapeutic approach when preoperative biopsy was not technically feasible, and there was a high suspicion of an invasive urinary tract tumor. Following radical nephroureterectomy, tissue samples were obtained for pathohistological and immunohistochemical analysis. The immunohistochemically analyzed cells were positive for leukocyte common antigen (LCA/CD45), vimentin (intermediate filament protein marker), cluster of differentiation 20 (CD 20, B-cell marker), CD79a (B-cell receptor-associated protein), paired box protein 5 (PAX5, B-cell lineage transcription factor), Ki67 (Ki-67 proliferation marker, 80%), B-cell lymphoma 6 protein (Bcl6, germinal center B-cell marker). Multiple myeloma oncogene 1 (MUM1/ IRF4, activated B-cell marker) showed partial positivity (<30%). The cells were negative for CD3 (T-cell marker), CD5 (T-cell/some B-cell marker), B-cell Lymphoma 2 protein(Bcl2, anti-apoptotic marker), CD10 (germinal center B-cell marker), Cyclin D1 (cell cycle regulatory protein), tumor suppressor protein (p63), and GATA binding protein 3 (GATA3, epithelial/urothelial marker) (Figure 2).

Through histopathological and immunohistochemical examination, the diagnosis of DLBCL, a subtype of NHL of the germinal center B-cell type (GCB subtype), was established. The classification of the GCB subtype was performed using the Hans algorithm based on the tumor’s immunohistochemical profile. Immunohistochemistry showed positivity for CD20, CD79a, PAX5, and Bcl6, with a high Ki-67 proliferative index (~80%), indicating aggressive tumor biology. The immunohistochemical profile, characterized by negativity for Bcl2 and CD10, positivity for Bcl6, and partial MUM1 expression (<30%), was consistent with the GCB subtype. Further staging work-up using MSCT of the chest and abdomen revealed no evidence of systemic disease dissemination. Therefore, the disease was classified as localized clinical stage I (extranodal involvement) CS-I AE. The absence of B symptoms, including unexplained fever, night sweats, and unintentional weight loss, was also confirmed. Pulmonary nodules were not involved by lymphoma, as excluded by the performed diagnostic work-up and radiological findings. Their clinical significance is that they most likely represented incidental benign reactive lesions without signs of malignant infiltration and therefore had no impact on disease staging or the choice of therapeutic approach.

After establishing the diagnosis, a multidisciplinary hematology team recommended initiation of treatment according to the R-CHOP protocol. R-CHOP is the standard treatment for DLBCL and is typically administered in six to eight cycles, depending on disease stage and risk assessment. Treatment response is evaluated clinically, radiologically, and through laboratory findings, with the goal of achieving complete or partial remission. The most common adverse effects of the R-CHOP regimen include myelosuppression (neutropenia, anemia, and thrombocytopenia), alopecia, nausea and vomiting, peripheral neuropathy (most often due to vincristine), cardiotoxicity (due to doxorubicin), and an increased risk of infections due to immunosuppression. Most of these adverse effects are reversible or manageable with appropriate supportive care and careful patient monitoring. The patient started immunochemotherapy, which she tolerated well without significant complications. After completion of the first treatment cycle, the patient was discharged in stable general condition with a recommendation to continue the planned chemotherapy cycles and undergo regular follow-up with a hematologist.

## Discussion

DLBCL is the most common subtype of NHL; however, involvement of the ureter is extremely rare. Due to this rare ureteral localization, DLBCL can present a significant diagnostic challenge because of its clinical and radiological similarity to ureteral urothelial tumors [6]. Very few cases of primary ureteral lymphoma have been described in the literature, and the exact incidence is difficult to estimate because of its extreme rarity [7-10]. The clinical presentation in our patient, initially suggestive of a ureteral tumor, is nonspecific for lymphoma and is consistent with previously reported cases in which urinary tract lymphomas mimic urothelial carcinoma or other causes of obstructive uropathy [11,12]. However, atypical presentations such as prominent hematuria or other acute urinary tract manifestations have also been described and may further obscure the underlying hematologic etiology. Even with the use of MSCT and other radiological methods, the imaging appearance of ureteral masses is difficult to distinguish from urothelial malignancies, often leading to inaccurate preoperative assessment [13].

Histopathological and immunohistochemical analyses remain crucial for establishing a definitive diagnosis, especially in unusual locations such as the ureter. The observed immunophenotype, positivity for CD20, CD79a, PAX5, and Bcl6, with a high Ki-67 index, indicates aggressive tumor biology characteristic of DLBCL [6,7]. This pattern aligns with previously reported cases of primary urinary tract lymphoma, including ureteral DLBCL [8,9]. Regarding DLBCL in the urinary tract, which includes the kidneys, bladder, ureters, and urethra, epidemiological analyses show that most cases affect the kidneys and bladder, while ureteral localization is extremely rare (<1%) [10,11]. Although clinically and radiologically indistinguishable from other tumors, urinary tract DLBCL shares similar histological features and behavior with other extranodal DLBCL subtypes, highlighting the need for detailed immunohistochemical evaluation of any atypical urological mass [12]. Treatment primarily involves a systemic chemotherapy regimen combined with rituximab (R-CHOP), considered the standard of care for DLBCL, which has significantly improved response rates and survival in many extranodal presentations [13]. In our case, the patient tolerated R-CHOP well without major adverse effects, consistent with clinical experience that early and adequate therapy contributes to favorable clinical outcomes and prognosis [6,7].

Compared with primary lymphomas in other parts of the urinary tract, such as the bladder or kidneys, these tumors also exhibit aggressive behavior and require a combination of chemotherapy and, in some cases, surgical intervention as part of the diagnostic and therapeutic approach [8,9]. Primary ureteral DLBCL is rare, and chemotherapy is often the first-line treatment, achieving complete remission when initiated promptly [10]. Although data on long-term outcomes of primary ureteral DLBCL are limited because of the small number of reported cases, registry analyses of primary urinary tract DLBCL have shown that combined surgical resection and systemic chemotherapy are associated with improved overall and disease-specific survival. Advanced age, higher disease stage, and the absence of systemic therapy have been identified as adverse prognostic factors in these studies [11,12].

A key limitation in diagnosing ureteral lymphoma is the difficulty of differentiating it from carcinoma before biopsy or surgery, often leading to radical nephroureterectomy before establishing the correct hematologic diagnosis. This underscores the need for early endoscopic biopsy whenever possible and for considering lymphoma in the differential diagnosis of unexplained ureteral masses [14,15]. Lymphoma should be particularly suspected when the ureteral lesion has an atypical appearance or location for urothelial carcinoma, when there is rapidly progressive obstruction without a clearly identifiable primary tumor mass, when there is disproportionate involvement of surrounding soft tissues or adjacent structures compared with the intraluminal finding, or when typical radiological features of urothelial tumors are absent. Additionally, clinical suspicion should be increased in patients presenting with systemic symptoms, enlarged lymph nodes, or laboratory findings suggestive of a hematologic disorder. Such an approach may improve clinical awareness, enable earlier diagnosis, reduce the risk of unnecessary radical surgical interventions, and allow timely initiation of appropriate therapy.

A limitation of this report is the absence of a preoperative biopsy, which precluded histopathological confirmation prior to surgical intervention and may have influenced the initial therapeutic approach, as well as the relatively short duration of patient follow-up, which limits the assessment of long-term outcomes, disease recurrence, and overall survival. European Society for Medical Oncology (ESMO) guidelines for aggressive B-cell lymphomas emphasize that tissue biopsy is mandatory for diagnosis and classification, as treatment with R-CHOP should not be initiated without histopathological confirmation [16].

## Conclusions

This case presents a rare localization of DLBCL in the ureter, which may mimic a primary urological tumor and significantly complicate the initial diagnosis. The definitive diagnosis was established only after surgical intervention and histopathological analysis. Although it is an aggressive lymphoma with a high proliferative index, the disease was detected at an early stage. Timely initiation of immunochemotherapy according to the R-CHOP protocol was well tolerated without significant complications. This clinical course emphasizes the importance of early diagnosis and an appropriate therapeutic approach in achieving a favorable outcome.

This case further highlights the clinical significance of recognizing lymphoma as a possible diagnosis in patients with ureteral masses and the need to include it in the differential diagnosis of urological tumors in order to avoid unnecessary delays in treatment. The clinical take-home message is that lymphoma should be suspected in atypical, rapidly progressive, or non-characteristic ureteral lesions, particularly when the clinical and radiological findings are not consistent with typical urological tumors, thereby enabling earlier diagnosis and timely initiation of therapy.
